# Salivary inflammatory biomarkers are predictive of mild cognitive impairment and Alzheimer’s disease in a feasibility study

**DOI:** 10.3389/fnagi.2022.1019296

**Published:** 2022-11-10

**Authors:** Kym McNicholas, Maxime François, Jian-Wei Liu, James D. Doecke, Jane Hecker, Jeff Faunt, John Maddison, Sally Johns, Tara L. Pukala, Robert A. Rush, Wayne R. Leifert

**Affiliations:** ^1^Molecular Diagnostic Solutions Group, Human Health Program, CSIRO Health and Biosecurity, Adelaide, SA, Australia; ^2^School of Biological Sciences, The University of Adelaide, Adelaide, SA, Australia; ^3^CSIRO Land and Water, Black Mountain Research and Innovation Park, Canberra, ACT, Australia; ^4^Australian e-Health Research Centre, CSIRO, Herston, QLD, Australia; ^5^Department of Internal Medicine, Royal Adelaide Hospital, Adelaide, SA, Australia; ^6^Department of General Medicine, Royal Adelaide Hospital, Adelaide, SA, Australia; ^7^Aged Care Rehabilitation and Palliative Care, SA Health, Modbury Hospital, Modbury, SA, Australia; ^8^School of Physical Sciences, The University of Adelaide, Adelaide, SA, Australia; ^9^Biosensis Pty Ltd., Adelaide, SA, Australia

**Keywords:** saliva, dementia, Alzheimer’s disease, cognitive impairment, inflammation, biomarker

## Abstract

Alzheimer’s disease (AD) is an insidious disease. Its distinctive pathology forms over a considerable length of time without symptoms. There is a need to detect this disease, before even subtle changes occur in cognition. Hallmark AD biomarkers, tau and amyloid-β, have shown promising results in CSF and blood. However, detecting early changes in these biomarkers and others will involve screening a wide group of healthy, asymptomatic individuals. Saliva is a feasible alternative. Sample collection is economical, non-invasive and saliva is an abundant source of proteins including tau and amyloid-β. This work sought to extend an earlier promising untargeted mass spectrometry study in saliva from individuals with mild cognitive impairment (MCI) or AD with age- and gender-matched cognitively normal from the South Australian Neurodegenerative Disease cohort. Five proteins, with key roles in inflammation, were chosen from this study and measured by ELISA from individuals with AD (*n* = 16), MCI (*n* = 15) and cognitively normal (*n* = 29). The concentrations of Cystatin-C, Interleukin-1 receptor antagonist, Stratifin, Matrix metalloproteinase 9 and Haptoglobin proteins had altered abundance in saliva from AD and MCI, consistent with the earlier study. Receiver operating characteristic analysis showed that combinations of these proteins demonstrated excellent diagnostic accuracy for distinguishing both MCI (area under curve = 0.97) and AD (area under curve = 0.97) from cognitively normal. These results provide evidence for saliva being a valuable source of biomarkers for early detection of cognitive impairment in individuals on the AD continuum and potentially other neurodegenerative diseases.

## Introduction

Alzheimer’s disease is a mostly sporadic, terminal neurodegenerative disease. The pathology of AD in the brain is distinctive. Plaques form outside neurons from accumulated deposition of insoluble, fibrillar amyloid-ß protein. Inside neurons, abnormal hyper-phosphorylated tau protein forms insoluble neurofibrillary tangles. Both tangles (Johnson et al., 2016) and plaques (Klunk et al., 2004) can be seen *in vivo* by positron emission tomography imaging and at post-mortem examination of the AD brain (Braak and Braak, 1991). This pathology forms over a considerable length of time (Jansen et al., 2015; McDade et al., 2018) with subjective cognitive decline often the first reported symptom (Albert et al., 2011). A key challenge is to detect this disease in the early pre-clinical stage before subtle changes in cognition occur.

Considerable progress has been made in the last 10 years in identifying AD-specific biomarkers in CSF and blood, with the focus being mainly on hallmark proteins, tau and amyloid-ß. However, early detection of AD biomarkers will require screening a wide group of healthy, asymptomatic individuals. Sample collection will need to be non-invasive, simple, and economical and saliva fits these criteria. Saliva can be self-collected and sampled repeatedly, as is evident with the success of the rapid antigen devices for coronavirus disease 2019 (COVID-19) with such tools likely to become more common with recent advances in biosensors (Goldoni et al., 2022). Saliva and the oral microbiome have the potential to be a rich source of biomarkers, containing over 5,000 proteins (Grassl et al., 2016) with promising results in the detection of colorectal (Lázaro-Sánchez et al., 2020), oral (Carnielli et al., 2018; Banavar et al., 2021) and prostate (Khan et al., 2018) cancers as well as systolic heart failure (Zhang et al., 2019).

Both AD biomarkers, tau and amyloid-β, have been found in saliva. Total tau protein is not altered in saliva of AD individuals (Lau et al., 2015; Ashton et al., 2018; Tvarijonaviciute et al., 2020) but more promisingly, the ratios of specific phosphorylated tau residues to total tau are reported to be significantly higher in AD saliva (Shi et al., 2011; Pekeles et al., 2019). Interestingly, levels of the amyloid-β_(1–42)_ peptide have been shown to increase in AD saliva (Bermejo-Pareja et al., 2010; Kim et al., 2014; Lee et al., 2017; Sabbagh et al., 2018; Katsipis et al., 2021), in contrast to the pattern of reduced levels observed in AD blood (Nakamura et al., 2018). Other promising AD biomarkers detected in saliva include glial fibrillary acidic protein (Katsipis et al., 2021), lactoferrin (Carro et al., 2017; González-Sánchez et al., 2020; Gleerup et al., 2021a; Reseco et al., 2021) and neuronal damage marker, neurofilament light chain (Gleerup et al., 2021b; Monroe et al., 2022).

A recent shotgun liquid chromatography-mass spectrometry (LC-MS) study reported alterations in proteins from multiple cellular pathways in saliva from individuals diagnosed with mild cognitive impairment (MCI) or AD (François et al., 2021). The aim of this work was to extend these promising findings by determining if the differences in these biomarkers in saliva could distinguish persons with MCI or AD from cognitively normal. Five proteins with key roles in inflammation, a pathway shown to be significantly altered in AD (François et al., 2021), were chosen for this work. The concentrations of Cystatin-C (CST-C), Interleukin-1 receptor antagonist (IL-1RN), Stratifin (SFN), Matrix metalloproteinase 9 (MMP-9) and Haptoglobin (HP) proteins were measured in saliva by enzyme-linked immunosorbent assay (ELISA). Receiver operating characteristic (ROC) analysis was used to show these proteins in saliva had the power to discriminate both AD and MCI from cognitively normal individuals.

## Methods

### Participants in South Australian neurodegenerative disease cohort

CSIRO, in collaboration with South Australian hospitals and clinicians, established the South Australian Neurodegenerative Disease cohort (SAND) database of saliva samples. Collection of patient saliva was approved by the Human Research Ethics Committee (CSIRO Ref 09/11). All participants were aged over 55 years and provided written informed consent before participating in this study. Saliva samples for this study were split into three cohorts; individuals clinically diagnosed with MCI (*n* = 15), individuals clinically diagnosed with AD (*n* = 16) and age and gender-matched cognitively normal (CN) controls (*n* = 29). Clinicians diagnosed patients based on criteria outlined by the National Institute on Aging-Alzheimer’s Association workgroups for AD (McKhann et al., 2011) and MCI (Albert et al., 2011) as reported previously (François et al., 2021). Patients with significant cognitive co-morbidities including head trauma, alcoholism, learning disability or Parkinson’s disease were excluded from the study.

### Saliva collection

All SANDs participants were asked to avoid eating and drinking before saliva collection. Saliva was collected using a collection device (RNAPro•SAL, Oasis Diagnostics Corporation^®^, Vancouver, WA, USA) following the manufacturer’s protocol. Briefly, an absorbent pad was placed in the participant’s mouth until the sample volume adequacy indicator fully changed color. The pad was then compressed by plunger into two collection tubes with a protein stabilizing solution (Oasis Diagnostics Corporation^®^, #PSS-001) added. All samples were stored at −80^°^C until assay completion.

### Total protein concentration in South Australian neurodegenerative disease cohorts saliva

The concentration of total salivary protein was measured by Bradford (Bio-Rad #5000006) and BCA (Thermofisher Pierce BCA #23227) assays following the manufacturers’ protocol. For both assays, three serial dilutions of each sample were measured. A pooled quality control saliva, protein standards and blanks were included on every 96-well plate.

### Mass spectrometry analysis of South Australian neurodegenerative disease cohorts saliva

The method for saliva preparation and shotgun LC-MS analysis of SANDs cohort samples has been previously described (François et al., 2021).

### Apolipoprotein E genotyping of South Australian neurodegenerative disease cohorts

Genotyping for *APOE* alleles ε2, ε3, ε4 in the SANDs blood samples used Real-Time PCR and TaqMan probes as previously described (François et al., 2021).

### Biomarker enzyme-linked immunosorbent assays

For the SFN ELISA, 96-well plates were incubated overnight at 4°C with capture antibody rabbit polyclonal to SFN (80 μL, 3 μg/mL, Biosensis, R-2115-100) in coating buffer pH 9.6. After washing, plates were blocked overnight at 4°C and air-dried before being vacuum sealed and stored at 4°C. On the day of use, a standard curve with seven points ranging from 50 ng/mL to 0.78 ng/mL was prepared using a full-length recombinant human SFN (ProSpec, #pka-357) in triplicate in sample buffer. Dilution linearity was achieved for SFN in saliva using a buffer containing two detergents, Triton-X (2%) and sodium deoxycholate (3%). Plates with standards and samples were incubated for 3 h. After washing, detection antibody mouse monoclonal to SFN clone 5D7 (100 μL, 250 ng/mL, Santa Cruz Biotechnology, sc-100638) was added and the plate incubated for 1 h. Following washing, horseradish peroxidase conjugated donkey anti-mouse IgG antibody (Jackson ImmunoResearch 711-035-151, 400 ng/mL) was added and incubated for 2 h before substrate was added.

MMP-9 was measured in saliva using a commercial ELISA kit (Biosensis, BEK-2073) following the manufacturer’s protocols with minor modifications. ELISA assays for measuring CST-C, IL-1RN and HP in saliva were developed using commercial antibody pairs (Human Cystatin C ELISA, Biosensis, BES-4010, Human IL-1RA ELISA, Biosensis, BES-4020 and Human Haptoglobin DuoSet ELISA R&D Systems, DY8465-05) as per the manufacturer’s instructions with some modifications. All assays were tested for dilution linearity in saliva and optimal dilution range before assaying patient samples.

On the day of use, plates and reagents were brought to room temperature. Saliva samples were randomly assigned to 96-well plates. Two serial dilutions of each saliva (two replicates) were measured. Standards, blank wells, and quality control saliva samples dilutions were included on every 96-well plate.

### Statistical methodology

The concentration of target in each sample was interpolated using 5-parameter logistic (5-PL) analysis. For the BCA assay, concentrations were interpolated using second-order quadratic regression analysis. Only saliva samples with coefficient of variation (CV) < 20% were included. To pass inter-assay variation requirements, only plates having quality control samples with CVs ≤ 10% were included in the analysis.

Biomarker data was log transformed prior to analyses. Outliers were removed using the Inter Quartile Range method and imputed with median values (no more than 2 per biomarker). Statistical comparisons of mean biomarker values per clinical group were performed using generalized linear models both uncorrected and corrected for gender, age and *APOE*ε4 allele status. Performance of target biomarkers to distinguish clinical groups was evaluated using receiver operating characteristic (ROC) curve analyses. Performance of an optimal multivariate panel of markers including age, gender and *APOE*ε4 allele status to predict clinical status, was compared with the performance of the base model (including age, gender and *APOE*ε4 allele status only) using the DeLong’s method (DeLong et al., 1988). To determine the degree of association between the ELISA and MS assays, Lin’s concordance correlation coefficients (Lin, 1989), Bland-Altman plots and Spearman rank Correlation tests were calculated. A Bonferroni corrected *p*-value < 0.001 (0.05/44 [number of tested performed]) was used for all comparisons. Where *p*-values were between the nominal significance level of 0.05 and the Bonferroni corrected significance level at <0.001, associated were classed as nominally significant. All calculations were carried out using SIMCA (version 16, Sartorius Stedim Biotech, Umeå, Sweden), the R Statistical Environment (Version 4.0.4) and GraphPad Prism (Version 9.0.0).

## Results

### Biomarker levels in saliva

Demographic information for participants from the SANDs cohort are summarized in Table 1 and Supplementary Table 1. Five proteins, CST-C, IL-1RN, SFN, MMP-9 and HP were chosen for further investigation from an earlier MS study because they were detected in all SAND cohort samples with significant differences in their relative abundance between the CN controls and AD and MCI cohorts (Supplementary Figure 1). In this work, all five targets were successfully detected by ELISA in all saliva samples with quality control performance data shown in Supplementary Table 2.

**TABLE 1:** Demographic data of SANDs cohort.

	Total	AD	MCI	CN	*P*-value
*N* (%)	60	16 (27%)	15 (25%)	29 (48%)	
Gender, M/F	32/28	10/6	7/8	15/14	0.662^†^
*APOE* ε4 carrier, *N* (%)	24 (40%)	9 (56%)	9 (60%)	6 (21%)	0.013^†^
Mean age (SD)	76 (7)	79 (6)	76 (6)	74 (8)	0.029^‡^
MMSE, median (MAD)	28 (3)	22 (3.7)	28 (1.5)	29 (1.5)	<0.0001*

^†^Chi-square test.

^‡^Ordinary ANOVA.

*Kruskal-Wallis ANOVA.

AD, Alzheimer’s disease; CN, Cognitively Normal; F, Female; MAD, Median absolute deviation; MCI, Mild cognitive impairment; MMSE, Mini-mental state examination; M, Male.

Overall, the total protein content of saliva from individuals, with either MCI or AD, was significantly higher compared to CN controls (Table 2). In a subset of SANDs samples (*n* = 36), levels of total protein were measured by an alternate method, BCA assay (Supplementary Table 3 and Supplementary Figure 2). Protein estimates from both Bradford and BCA assays closely agreed with a concordance coefficient of 0.97 [95% CI 0.95–0.99], confirming that protein was indeed significantly higher in the saliva of individuals with MCI or AD as measured by two protein assays with differing chemistry.

**TABLE 2:** Univariate model analysis of biomarkers in SAND cohort saliva.

Biomarker	CN vs. MCI	CN vs. AD	MCI vs. AD	CN vs. MCI/AD
Total protein (μg/mL)^†^	<0.0001	0.02	0.1	<0.0001
CST-C (μg/mL)^†^	0.02	0.26	0.01	0.50
CST-C/total protein ratio^†^	0.07	0.002	0.28	0.0008
IL-1RN (μg/mL)^†^	0.01	0.63	0.02	0.32
IL-1RN/total protein ratio^†^	0.30	0.01	0.17	0.03
SFN (μg/mL)^†^	0.03	0.55	0.03	0.48
SFN/total protein ratio^†^	0.10	0.04	0.67	0.02
MMP-9 (μg/mL)^†^	0.04	0.0002	0.50	0.0003
MMP-9/total protein ratio^†^	0.44	0.002	0.11	0.02
Hp (μg/mL)^†^	0.003	0.02	0.51	0.0007
Hp/total protein ratio^†^	0.21	0.08	0.41	0.06

^†^Unadjusted *p*-values shown from log transformed data of absolute concentrations and their ratio to total protein.

AD, Alzheimer’s disease; CN, Cognitively Normal; CST-C, Cystatin-C; Hp, Haptoglobin; IL-1RN, Interleukin-1 receptor antagonist protein; Matrix metalloproteinase 9, MMP-9; MCI, Mild cognitive impairment; SFN, Stratifin; vs., versus.

**FIGURE 1:**
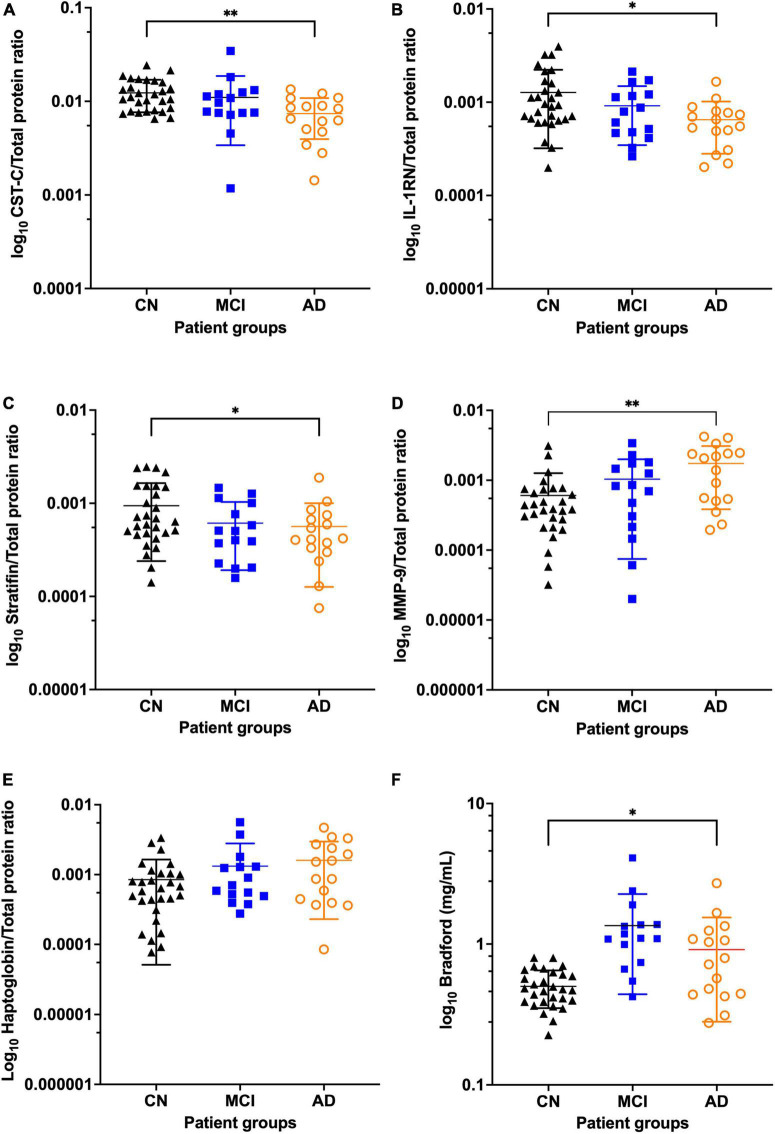
Levels of total protein and five biomarkers in the SANDs cohort showing reduced abundance of CST-C, IL-1RN, and SFN in MCI and AD and increased abundance of total protein, MMP-9 and HP in MCI and AD. Scatter dot plots show the mean, standard deviation and all points for each biomarker adjusted for total protein with AD in orange (*n* = 16), MCI in blue (*n* = 15) and CN in black (*n* = 29) for **(A)** CST-C **(B)** IL-1RN **(C)** SFN **(D)** MMP-9 **(E)** Hp **(F)** Total protein. *Unadjusted *p*-values shown from log transformed data for cohorts CN vs. AD with *P < 0.05 and ^**^P < 0.01. AD, Alzheimer’s disease; CN, Cognitively Normal; CST-C, Cystatin-C; Hp, Haptoglobin; IL-1RN, Interleukin-1 receptor antagonist protein; Matrix metalloproteinase 9, MMP-9; MCI, Mild cognitive impairment; SFN, Stratifin.

In this study, the concentrations of targets measured by ELISA were adjusted for total salivary protein so that comparisons were comparable with the earlier MS data. Overall, three targets, CST-C, IL-1RN and SFN, showed a pattern of reduced abundance in MCI and AD saliva whereas levels of MMP-9 and HP increased (Figure 1 and Supplementary Table 4). In saliva from participants with MCI, absolute levels of all five targets were nominally significantly different from saliva of the CN controls although this significance dropped when adjusted for total protein (Table 2). In saliva from individuals diagnosed with AD, four out of five targets as a proportion of total protein showed a significant difference compared to age and sex matched CN controls with two targets remaining statistically significant after post correction for multiple comparisons (*P* < 0.001) (Table 2). In the comparison between controls and cognitive impairment overall (CN vs. MCI/AD), all five targets were nominally significantly altered (Table 2) with two targets remaining statistically significant after post correction for multiple comparisons, supporting the finding that the abundance of these proteins were altered by disease progression. *Post hoc* power analysis (using sample size, two-tailed alpha = 0.05) shows that both CST-C and MMP-9, adjusted for total protein, had greater than 90% power to detect statistically significant differences between CN and AD. Similar analysis shows that total salivary protein had 98% power to detect a significant difference between CN and MCI and targets, HP and CST-C, had 80% power.

To evaluate the agreement between the earlier MS data and the concentrations measured by ELISA, concordance correlation coefficients were calculated for each target. Four of the targets demonstrated good agreement between the two assays [MMP9 0.75 (95% CI 0.62–0.85), CST-C 0.78 (95% CI 0.66–0.87), IL-1RN 0.87 (95% CI 0.79–0.92), HP 0.84 (95% CI 0.84–0.90)]. SFN was the exception with only a weak agreement [0.21 (95% CI -0.044 to 0.44)]. Bland-Altman plots graphically show the agreement between two assays with the bias close to zero and no obvious trends in data points above or below (Supplementary Figure 3). Similarly, Spearman correlations found strong agreement for four targets and slightly weaker for SFN (Supplementary Figure 4).

### Saliva biomarkers as predictors of mild cognitive impairment and Alzheimer’s disease

**FIGURE 2:**
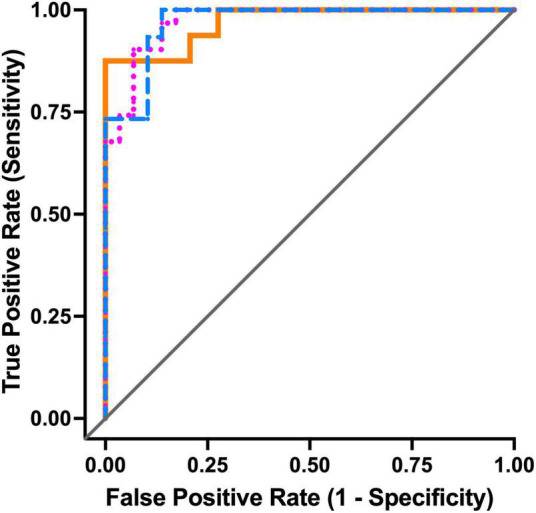
Receiver operating characteristic curve analyses for the top three models. In the comparison CN vs. MCI (blue dashed line), AUC = 0.97 and top model is base model with SFN (μg/mL) and total protein (μg/mL), accuracy = 91%. For CN vs. AD (orange solid line), AUC = 0.97 and top model is base model with CST-C (total protein ratio) and IL-1RN (total protein ratio), accuracy = 96%. For CN vs. combined MCI/AD (pink dotted line), AUC = 0.97 and top model is base model with CST-C (total protein ratio), IL-1RN (total protein ratio), MMP-9 (total protein ratio) and total protein (μg/mL), accuracy = 92%. AD, Alzheimer’s disease; CN, Cognitively Normal; CST-C, Cystatin-C; IL-1RN, Interleukin-1 receptor antagonist protein; Matrix metalloproteinase 9, MMP-9; MCI, Mild cognitive impairment; SFN, Stratifin.

ROC analysis (Figure 2) showed that for the comparison between CN controls and MCI participants, a combined model of SFN (μg/mL), total protein (μg/mL) and the base model confounders age, gender and *APOE* ε4 allele status showed excellent sensitivity and specificity with an AUC of 0.97 (95%CI 0.93–1.00), a significant improvement (*p* < 0.0001) on the base model alone with an AUC of 0.75 (95%CI 0.60–0.91). The combination of base model with CST-C (total protein ratio) and IL-1RN (total protein ratio) also showed excellent performance in predicting those participants with AD compared with CN controls [AUC = 0.97, (95%CI 0.92–1.00)]. Overall, a panel of markers that includes the base model and CST-C (total protein ratio), IL-1RN (total protein ratio), MMP-9 (total protein ratio) and total protein (μg/mL) could distinguish either MCI or AD from the CN controls with an AUC = 0.97 (95%CI 0.94–1.00).

## Discussion

This work sought to extend the promising findings of a recent MS study in saliva from individuals diagnosed with MCI or AD and CN. Pathway analysis in the previous work showed that levels of inflammatory markers were significantly altered in AD (François et al., 2021). All five proteins chosen in this study have key roles in inflammation pathways. IL-1RN is a member of the interleukin-1 family of signaling cytokines. It plays an anti-inflammatory role by inhibiting the activity of major pro-inflammatory cytokines (interleukin-1 α/β) by binding to the IL-1 receptor thereby blocking further signaling and dampening IL-1 mediated inflammation (Dripps et al., 1991). SFN, also known as 14-3-3 sigma, is a member of the highly conserved 14-3-3 family with important roles in DNA damage repair, cell-cycle arrest, apoptosis (Hermeking, 2003) and inflammation (Munier et al., 2021). SFN and IL-1RN are expressed in the squamous epithelial cells of the oral mucosa, the mucous membrane lining the mouth (Uhlen et al., 2015).^1^ Both SFN (Hu et al., 2005; Fang et al., 2007; Denny et al., 2008; Giusti et al., 2010; Krief et al., 2012, 2019; Salazar et al., 2013) and IL-1RN (Denny et al., 2008; Rao et al., 2009; Salazar et al., 2013; Dominy et al., 2014; Aziz et al., 2015; Twal et al., 2016; Xiao et al., 2016; Murr et al., 2017) proteins have been detected in saliva studies previously.

The multiple functions of CST-C include inhibition of cysteine proteases, host defense against pathogens, apoptosis, as well as roles in inflammatory diseases such as rheumatoid arthritis (Zi and Xu, 2018). CST-C is highly expressed by the salivary glands (Paraoan et al., 2010), in particular the submandibular and parotid glands (Saitou et al., 2020) and has been reported in numerous studies in saliva (Denny et al., 2008; Rao et al., 2009; Salazar et al., 2013; Ngounou Wetie et al., 2015). MMP-9 is a gelatinase in the matrix metalloproteinase family, essential proteolytic enzymes in wound healing and bone remodeling. MMP-9 is found in the major salivary glands, sublingual and submandibular (Saitou et al., 2020), secreted by immune cells such as neutrophils in response to inflammation (Song et al., 2013) and has also been detected in many saliva studies (Denny et al., 2008; Rao et al., 2009; Jaedicke et al., 2012; Salazar et al., 2013; Dominy et al., 2014; Costantini et al., 2020; Bostanci et al., 2021). HP is described as an acute-phase response protein stimulated by inflammatory cytokines such as TNF-alpha and interleukins IL-1 and IL-6. HP binds to circulating hemoglobin thereby avoiding toxicity of free hemoglobin and has been detected before in saliva (Denny et al., 2008; Rao et al., 2009; Haigh et al., 2010; Salazar et al., 2013; Ngounou Wetie et al., 2015; Murr et al., 2017; Tvarijonaviciute et al., 2020) with significantly higher levels in individuals with oral lichen planus (Mateo et al., 2019), a chronic oral inflammatory condition.

Only a few studies report total protein in saliva from individuals with MCI or AD. Three recent studies show a trend of increasing total protein in saliva from individuals diagnosed with MCI or AD (Galindez et al., 2021; Gleerup et al., 2021a,b). Higher levels of salivary protein have also been reported in saliva from individuals with Parkinson’s disease (PD) (Devic et al., 2011; Al-Nimer et al., 2014; Kang et al., 2014; Fedorova et al., 2015; Masters et al., 2015; Galindez et al., 2021) and have been attributed to reduced saliva flow (Fedorova et al., 2015) and PD related salivary gland dysfunction (Masters et al., 2015). Saliva flow rates decline with aging due to reduced secretions from two major salivary glands (Affoo et al., 2015). As the controls in this work were age- and gender- matched, age-related changes to salivary gland function are likely to affect all samples equally. Reduced saliva flow is associated with declining cognition (Sørensen et al., 2018; Do et al., 2021) so it is possible that in the individuals with cognitive impairment, saliva flows are further reduced leading to a concentration of proteins in their saliva.

While all five proteins have been found in numerous saliva studies, only a few shed light on why they may be altered in AD. CST-C is encoded by the *CST3* gene, considered to be a “risk” gene for AD (Bertram et al., 2007; Wang et al., 2021) with altered levels of this cystatin reported in both CSF and blood of AD individuals (Zhong et al., 2013; Wang et al., 2017; Chen et al., 2022). Abnormal levels of metalloproteinases, including MMP-9, in plasma have been associated with AD progression (Iturria-Medina et al., 2016) and increased permeability of the blood-brain barrier, a driver of cognitive impairment (Montagne et al., 2020). AD individuals have significantly higher plasma levels of HP (Zhu et al., 2018; Chen and Xia, 2020) but as yet there are no reports of altered HP in either MCI or AD saliva. Members of the 14-3-3 family have been linked to neurodegenerative diseases such as Parkinson’s (Wang et al., 2018) and sporadic Creutzfeldt–Jakob (Chohan et al., 2010) disease. The isoform 14-3-3 gamma is altered in the CSF of AD patients (Sathe et al., 2019; Falgàs et al., 2020) but there are no published reports of abnormal levels of 14-3-3 proteins in the saliva of individuals with AD or MCI. Epigenetic inactivation (hyper-methylation) of SFN has been associated with cancer (Yi et al., 2009) and periodontal disease (Wang et al., 2014).

Indeed, peripheral inflammatory diseases such as periodontitis have been linked with AD (Dioguardi et al., 2020; Liccardo et al., 2020; Hajishengallis and Chavakis, 2021; Xie et al., 2021). This association has been attributed to increased systemic inflammation, resulting from both the infiltration of periodontal pathogens and the inflammatory process of peripheral diseases like periodontitis, triggering the neuroinflammation of AD. Whether poor mouth health is a result of impaired cognition or a causative factor for declining cognition is not clear. Future saliva studies should record details on patients’ mouth health, co-morbidities, flow rates and include an analysis of the microbiome in these saliva samples, to find possible associations between the targets found in this study, periodontal disease pathogens and AD.

A limitation of the current study is the small number of samples tested (*n* = 60). Small sample sets can inflate the size of an effect (Button et al., 2013) and so further work is needed to validate these promising targets in saliva from a much larger cohort alongside neuroimaging data, conversion to AD from MCI data, and biofluid measurements of amyloid-β and tau, to assess the strength of these findings against these well validated AD biomarkers.

To our knowledge, there have only been two studies reporting on proteins in saliva from AD individuals using untargeted MS. In a small sample set (*n* = 6), Eldem et al. (2022) also found all five target proteins in their saliva but no significant difference in abundance was reported. Only CST-C was reported by Contini et al. (2021) with no difference in AD saliva compared to controls. Both studies used passive unstimulated saliva whereas in the current study, saliva was collected using a commercial device (RNAPro•SAL™). As the method of collection can change the protein profile of saliva (Topkas et al., 2012; Marksteiner et al., 2022), further work is needed to determine what effect, if any, this particular collection device had on levels of these targets and others in saliva. This knowledge would aid in the reproducibility of future studies and guide best practices for sample collection.

The abundance of proteins in multiple cellular processes and pathways are altered in the saliva of individuals with MCI or AD (François et al., 2021). In agreement with this earlier MS study, this work found five proteins in the saliva of individuals with MCI or AD had a consistent pattern of altered abundance as measured by ELISA. All five targets were detected in 100% of samples highlighting the utility of measuring proteins in saliva by ELISA. While MS is suited to biomarker discovery, ELISAs are routinely used in research and clinical settings as a useful tool for mass screening of samples for a biomarker because they are simple, economical and accessible. ROC analysis shows that combinations of these proteins in saliva demonstrate excellent diagnostic accuracy for detecting both MCI and AD. This is a significant finding in the quest for early detection in individuals on the AD continuum and other neurodegenerative diseases. It is not difficult to foresee a future where asymptomatic individuals could undertake regular, self-collected saliva testing and which gave an “early warning” to medical professionals of declining cognition and neurodegeneration. As a screening tool in primary care settings, medical professionals could then direct these individuals toward more specific testing and with early intervention, opening the possibility of halting and even reversing disease progression.

## Data availability statement

The original contributions presented in this study are included in the article/Supplementary material, further inquiries can be directed to the corresponding author/s.

## Ethics statement

The studies involving human participants were reviewed and approved by the Human Research Ethics Committee CSIRO Ref 09/11. The patients/participants provided their written informed consent to participate in this study.

## Author contributions

KM: study design, data collection, data interpretation, and writing. MF and WL: SANDs cohort design and collection, study design, data interpretation, and writing. JD: statistician, data interpretation, and writing. TP and RR: study design, data interpretation, and writing. J-WL: SANDs cohort mass spectrometry design and analysis. JH, JF, JM, and SJ: clinical input. All authors contributed to the article and approved the submitted version.

## Footnotes

1
https://www.proteinatlas.org
