# Supplementary material for: Salivary inflammatory biomarkers are predictive of mild cognitive impairment and Alzheimer’s disease in a feasibility study
Source: Front Aging Neurosci. 2022 Nov 10;14:1019296. doi: 10.3389/fnagi.2022.1019296 (PMC9685799; doi:10.3389/fnagi.2022.1019296)
Supplement: Supplementary file 1 [file Data_Sheet_1.zip › Figure2.docx]

Supplementary Figure 2.


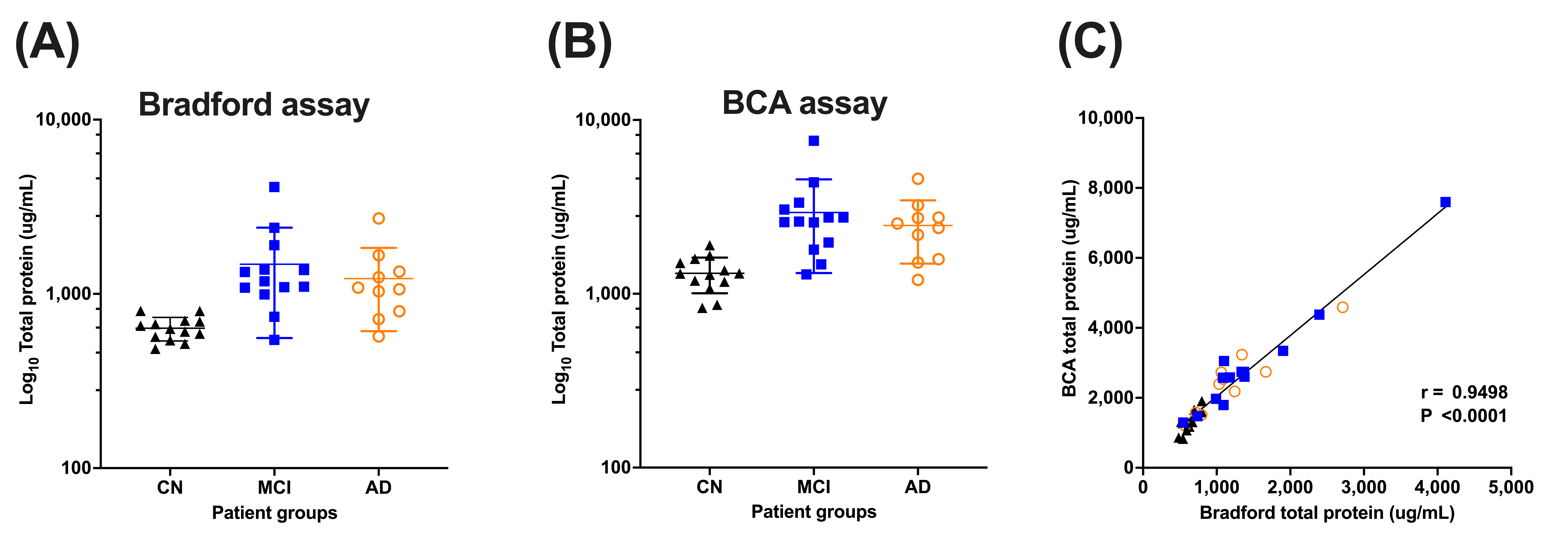
Supplemental Figure 2: Bradford and BCA assays both show increased levels of total protein in disease cohorts. Scatter dot plots showing mean and standard deviation for total protein estimations in the SANDs saliva cohort by **(A)** Bradford **(B)** BCA assays and **(C)** Spearman Correlation (r) between Bradford and BCA assays. AD in orange (n=10), MCI in blue (n=13) and CN in black (n=13). Abbreviations: AD, Alzheimer’s disease; BCA, Bicinchoninic acid assay; CN, Cognitively Normal; MCI, Mild cognitive impairment.
