# Supplementary material for: Salivary inflammatory biomarkers are predictive of mild cognitive impairment and Alzheimer’s disease in a feasibility study
Source: Front Aging Neurosci. 2022 Nov 10;14:1019296. doi: 10.3389/fnagi.2022.1019296 (PMC9685799; doi:10.3389/fnagi.2022.1019296)
Supplement: Supplementary file 1 [file Data_Sheet_1.zip › Figure3.docx]

Supplementary Figure 3. Bland-Altman plots



Supplemental Figure 3. Bland-Altman plots displaying the agreement between the ELISA and MS assays, for measurement of (**A)** CST-C **(B)** IL-1RN **(C)** SFN **(D)** MMP-9 and **(E)** Hp. Values for both assays were scaled. The solid black line represents the average bias, or average of differences. The upper and lower red dashed lines represent the 95% limits of agreement. Abbreviations: AD, Alzheimer’s disease; BCA, Bicinchoninic acid assay; CN, Cognitively Normal; CST-C, Cystatin-C; Hp, Haptoglobin; IL-1RN, Interleukin-1 receptor antagonist protein; MS, mass spectrometry; Matrix metalloproteinase 9, MMP-9; MCI, Mild cognitive impairment; SFN, Stratifin.
