# Supplementary material for: Salivary inflammatory biomarkers are predictive of mild cognitive impairment and Alzheimer’s disease in a feasibility study
Source: Front Aging Neurosci. 2022 Nov 10;14:1019296. doi: 10.3389/fnagi.2022.1019296 (PMC9685799; doi:10.3389/fnagi.2022.1019296)
Supplement: Supplementary file 1 [file Data_Sheet_1.zip › Figure4.docx]

Supplementary Figure 4. Spearman correlations plots



Supplemental Figure 4. Correlation plots of MS peptide abundance versus ELISA target concentrations (as a ratio to total protein) showing Spearman’s rank correlation (r) and p-value for SANDs saliva samples (n=60) with AD in orange (n=16), MCI in blue (n=15) and CN in black (n=29). (**A)** CST-C **(B)** IL-1RN **(C)** SFN **(D)** MMP-9 **(E)** Hp. Abbreviations: AD, Alzheimer’s disease; CN, Cognitively Normal; CST-C, Cystatin-C; Hp, Haptoglobin; IL-1RN, Interleukin-1 receptor antagonist protein; MS, mass spectrometry; Matrix metalloproteinase 9, MMP-9; MCI, Mild cognitive impairment; SFN, Stratifin.
