# Supplementary material for: Salivary inflammatory biomarkers are predictive of mild cognitive impairment and Alzheimer’s disease in a feasibility study
Source: Front Aging Neurosci. 2022 Nov 10;14:1019296. doi: 10.3389/fnagi.2022.1019296 (PMC9685799; doi:10.3389/fnagi.2022.1019296)
Supplement: Supplementary file 1 [file Data_Sheet_1.zip › Table1.docx]

Supplementary Table 1**:** Analysis of MMSE score between cohorts

|  | **AD vs CN** | **AD vs MCI** | **MCI vs CN** |
| --- | --- | --- | --- |
| **MMSE** | <0.0001 | <0.0001 | 0.03 |

Supplementary Table 1**:** p-values from unpaired two-tailed t-test. Abbreviations: AD, Alzheimer’s disease; CN, Cognitively Normal; MCI, Mild cognitive impairment; MMSE, Mini-mental state examination.
