# Supplementary material for: Salivary inflammatory biomarkers are predictive of mild cognitive impairment and Alzheimer’s disease in a feasibility study
Source: Front Aging Neurosci. 2022 Nov 10;14:1019296. doi: 10.3389/fnagi.2022.1019296 (PMC9685799; doi:10.3389/fnagi.2022.1019296)
Supplement: Supplementary file 1 [file Data_Sheet_1.zip › Table2.docx]

Supplementary Table 2**:** Quality Control performance of Bradford and ELISA assays

| **Assay** | **Dilution range** | **Inter-assay variation (%)** |
| --- | --- | --- |
| **Total protein*** | 1:2-1:64 | 1% |
| **CST-C** | 1:400-12,800 | 8% |
| **IL-1RN** | 1:400-1:6,400 | 2% |
| **SFN** | 1:40-1:320 | 10% |
| **MMP-9** | 1:35-1:2,000 | 5% |
| **Hp** | 1:400-1:12,800 | 6% |

Supplementary Table 2**:** All saliva samples had two serial dilutions within the dilution range for each target shown in this table. Quality control saliva samples were included on every plate and the coefficient of variation percentage for all quality control samples for each assay are shown.*from Bradford Assay
