# Supplementary material for: Salivary inflammatory biomarkers are predictive of mild cognitive impairment and Alzheimer’s disease in a feasibility study
Source: Front Aging Neurosci. 2022 Nov 10;14:1019296. doi: 10.3389/fnagi.2022.1019296 (PMC9685799; doi:10.3389/fnagi.2022.1019296)
Supplement: Supplementary file 1 [file Data_Sheet_1.zip › Table3.docx]

Supplementary Table 3**:** Total salivary protein for each cohort by protein estimation assay

|  | **AD (n=10)** | | **MCI (n=13)** | | **CN (n=13)** | | **p-value*** |
| --- | --- | --- | --- | --- | --- | --- | --- |
|  | **Mean** | **SD** | **Mean** | **SD** | **Mean** | **SD** |  |
| **Bradford (µg/mL)** | 1,223 | 612 | 1,479 | 920 | 635 | 98 | 0.0002 |
| **BCA (µg/mL)** | 2,470 | 979 | 2,935 | 1,620 | 1,311 | 304 | 0.0002 |

Supplementary Table 3**:** *p-value from Kruskal-Wallis ANOVA. Abbreviations: AD, Alzheimer’s disease; CN, Cognitively Normal; MCI, Mild cognitive impairment.
