# Supplementary material for: Salivary inflammatory biomarkers are predictive of mild cognitive impairment and Alzheimer’s disease in a feasibility study
Source: Front Aging Neurosci. 2022 Nov 10;14:1019296. doi: 10.3389/fnagi.2022.1019296 (PMC9685799; doi:10.3389/fnagi.2022.1019296)
Supplement: Supplementary file 1 [file Data_Sheet_1.zip › Table4.docx]

Supplementary Table 4**:** Absolute values for total protein and targets for each cohort

|  | **AD (n=16)** | | **MCI (n=15)** | | **CN (n=29)** | |
| --- | --- | --- | --- | --- | --- | --- |
|  | **Mean** | **SD** | **Mean** | **SD** | **Mean** | **SD** |
| **Total protein (µg/mL)** | 913 | 631 | 1354 | 915 | 500 | 149 |
| **CST-C (µg/mL)** | 6.97 | 7.08 | 13.67 | 11.95 | 5.89 | 2.21 |
| **CST-C/total protein ratio** | 0.00739 | 0.00344 | 0.01102 | 0.00763 | 0.01232 | 0.00468 |
| **IL-1RN (µg/mL)** | 0.63 | 0.57 | 1.05 | 0.66 | 0.64 | 0.52 |
| **IL-1RN/total protein ratio** | 0.00065 | 0.00037 | 0.00092 | 0.00057 | 0.00127 | 0.00095 |
| **SFN (µg/mL)** | 0.49 | 0.47 | 0.67 | 0.41 | 0.46 | 0.38 |
| **SFN/total protein ratio** | 0.00056 | 0.00044 | 0.00061 | 0.00042 | 0.00094 | 0.00070 |
| **MMP-9 (µg/mL)** | 1.55 | 1.80 | 1.48 | 1.75 | 0.31 | 0.38 |
| **MMP-9/total protein ratio** | 0.00174 | 0.00135 | 0.00104 | 0.00096 | 0.00061 | 0.00066 |
| **Hp (µg/mL)** | 1.69 | 2.18 | 1.98 | 2.31 | 0.44 | 0.52 |
| **Hp/total protein ratio** | 0.00160 | 0.00137 | 0.00132 | 0.00147 | 0.00085 | 0.00080 |

Supplementary Table 4**:** Absolute concentrations before outliers were removed and replaced with median values. Abbreviations: AD, Alzheimer’s disease; CN, Cognitively Normal; MCI, Mild cognitive impairment.
